# Supplementary material for: Manipulating topological transformations of polar structures through real-time observation of the dynamic polarization evolution
Source: Nat Commun. 2019 Oct 25;10:4864. doi: 10.1038/s41467-019-12864-5 (PMC6814840; doi:10.1038/s41467-019-12864-5)
Supplement: Supplementary file 1 — Supplementary Information [file 41467_2019_12864_MOESM1_ESM.pdf]

Supplementary Information for

Manipulating topological transformations of polar structures through real-time observation of the dynamic polarization evolution

Du et al.

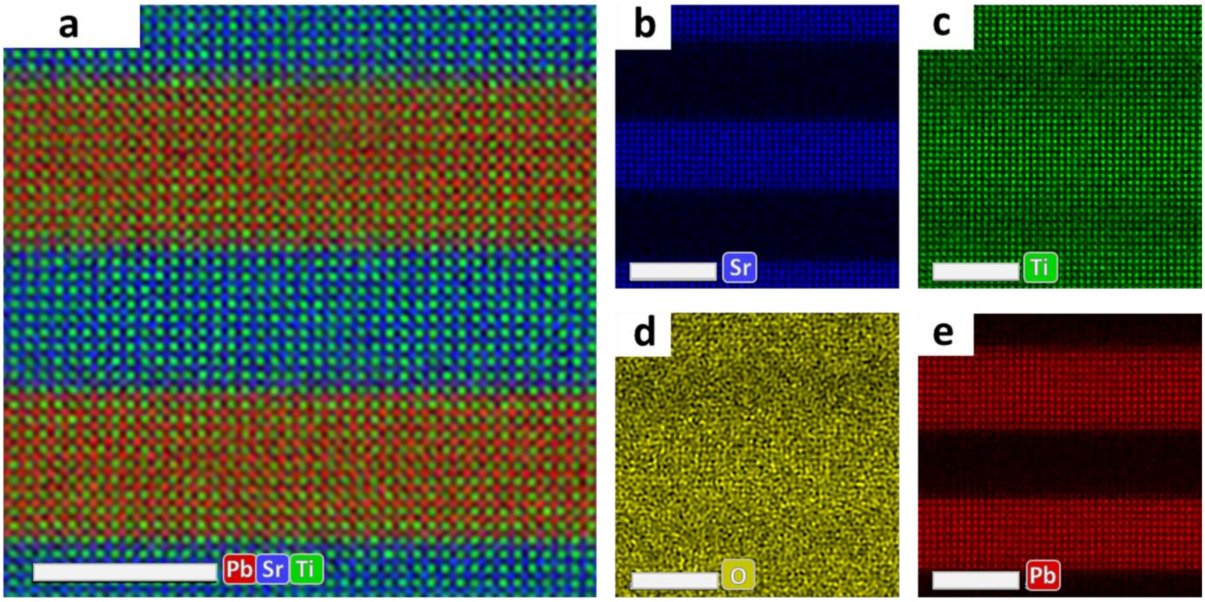

Supplementary Figure 1 EDS-mapping of Sr, Ti, O and Pb. **a** is the overlap of Pb, Sr and Ti elements. **b-e** is corresponding to Sr, Ti, O and Pb. Those images show that the interface of $PbTiO_3/SrTiO_3$ (PTO/STO) is atomically sharp. Due to the distortion of the oxygen octahedra, the column effect of the oxygen atoms is reduced in STEM mode; thus a slightly darker contrast in the O map at the position of the PTO layer is to be expected. Scale bar, 5 nm.

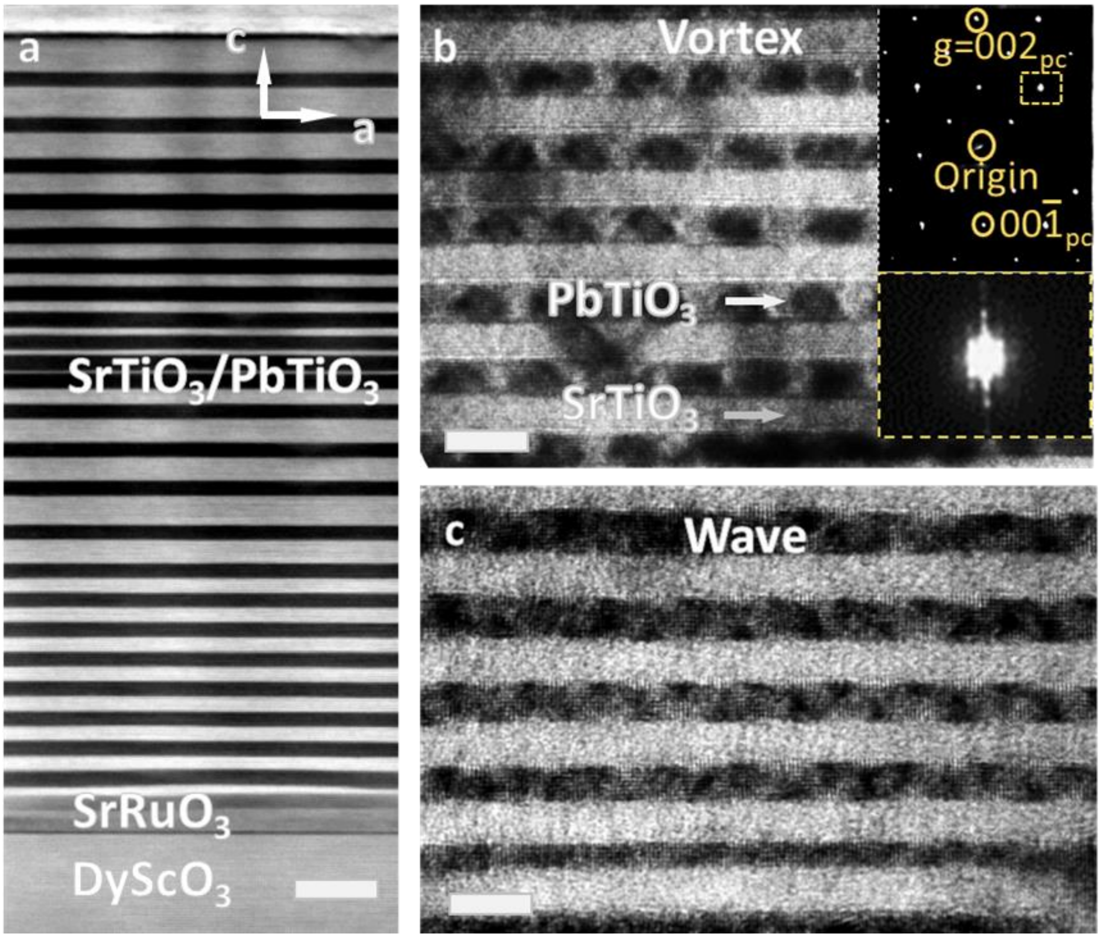

Supplementary Figure 2 Structural characterization of the $PTO_{(n)}/STO_{(10)}$ multilayer and long-range ordering of the polar structure. **a** Low-magnification STEM image of the cross-section of a $PTO_{(n)}/STO_{(10)}$ multilayer. Scale bar, 20 nm. **b** and **c** Cross-section DF-TEM images of a $PTO_{(n)}/STO_{(10)}$ multilayer revealing long-range ordering of vortices (**b**) and wave (**c**); each bright/dark modulation corresponds to a period of the clockwise-counterclockwise vortices structure. Inset, SAED pattern of the sample with the g-vector of the two-beam imaging condition indicated by the yellow circle. In a blow up of the reflection, weak additional spots are observed, resulting from the long-range ordering of topological structures, with the periodicity of ~9 nm which is in consistent with the periodicity of vortex configuration and the reports in Ref. 1. Scale bar, 5 nm.

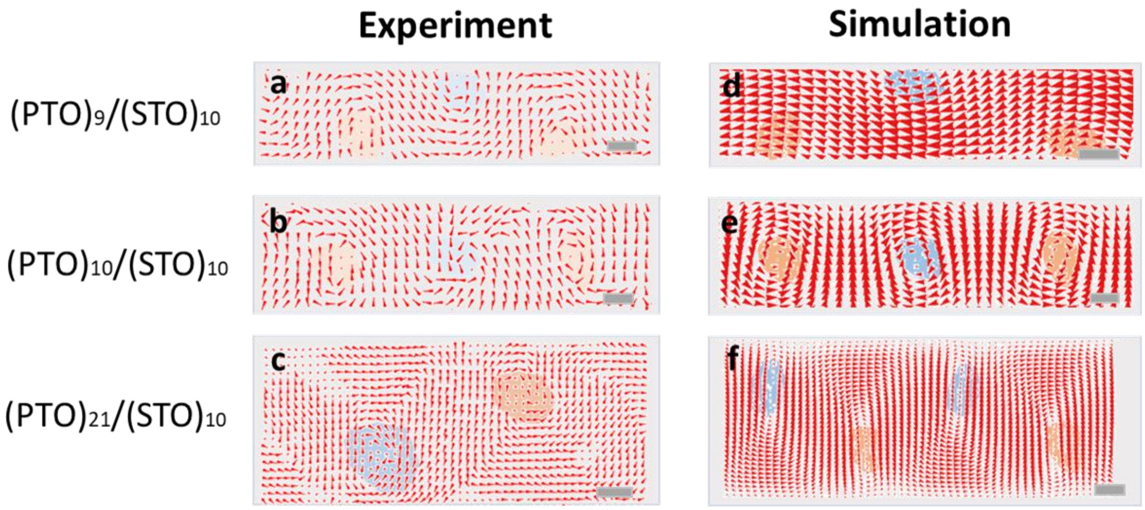

Supplementary Figure 3 Experiment and simulation results of the domain pattern as function of the PTO thickness. **a** and **d** for a wave structure at a PTO layer of 9 uc; **b** and **e** for a vortex structure in a PTO layer of 10 uc. Scale bar, 1 nm. **c** and **f** for a flux closure structure for a PTO layer of 21 uc. Scale bar, 2 nm.

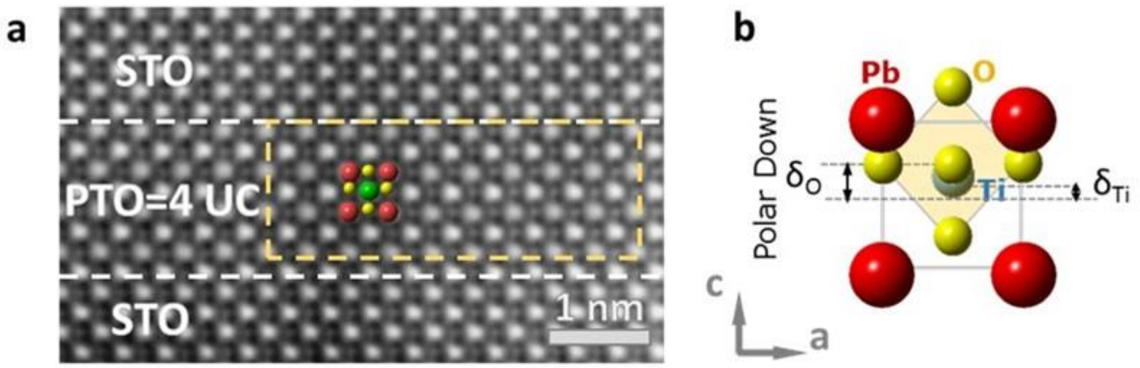

Supplementary Figure 4 The measurement of spontaneous polarization in local region based on iDPC images. **a** Atomic-level cross-section iDPC image of a 4 uc PTO layer superimposed with the structure model of $PbTiO_3$. In the room temperature tetragonal PTO structure, Ti^{4+} shifts relative to the Pb sublattice, while the oxygen octahedra shift in the same direction but with a larger displacement. According to the position of Ti and O atoms, we calculated the spontaneous polarization of some unit cells (yellow rectangle). The spontaneous polarization value of the PTO (4 uc) is 63.63 ± 3 μC/cm^2, close to that of bulk PTO (≈75 μC/cm^2). **b** Unit-cell projection of tetragonal PTO along the crystallographic b-axis. The displacement of Ti and O atoms with respect to the center of the Pb atoms along the c-axis are denoted as δ_{Ti} and δ_O.

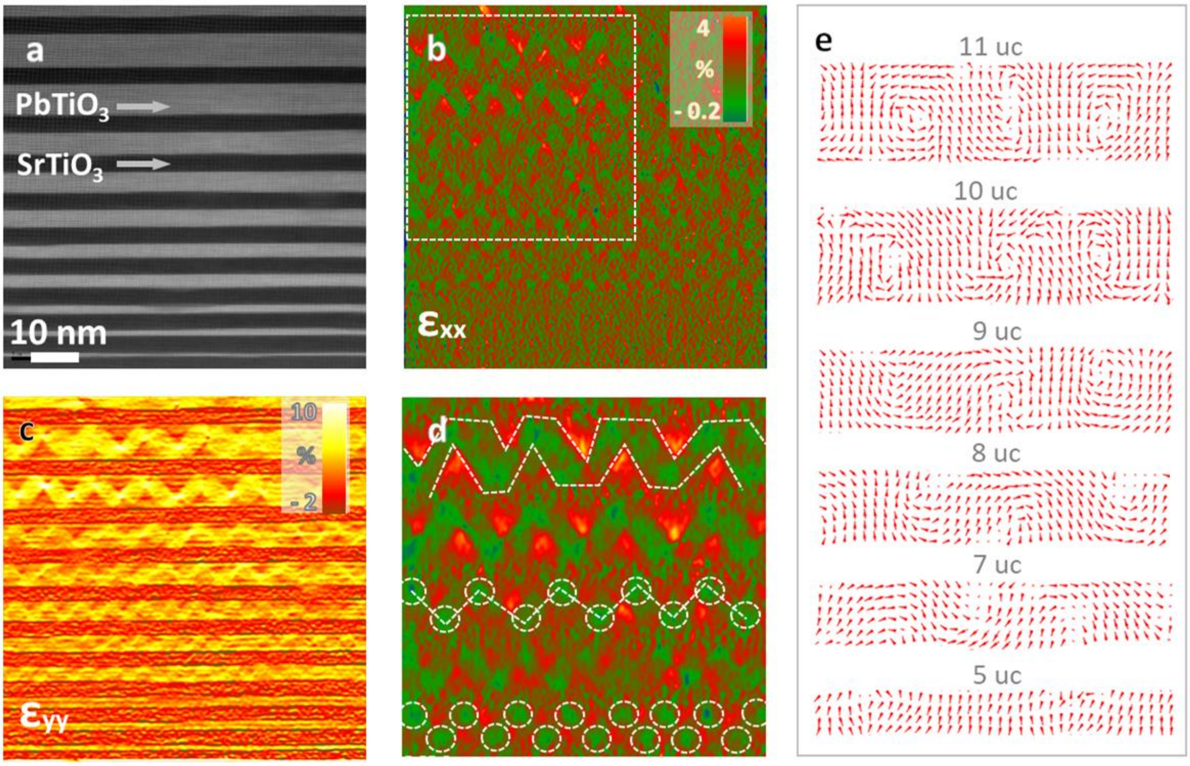

Supplementary Figure 5 In plane (ε_{xx}) and out-of-plane strain (ε_{yy}) maps. **a** is low-magnification STEM images of the cross-section of a $PTO_{(n)}/STO_{(10)}$ multilayer, with n=1-21. **b-c** are extracted via the geometric phase analysis (GPA). The strain shown displays a clear spatial arrangement related to the domain patterns. **d** GPA analysis of the STEM data extracted from white box in (**b**) showing that ε_{xx} also exhibits a periodicity characteristic, which is added with some marker lines to make the characteristic more visible. **e** As to acquire a better understanding of the transition state between vortex and wave, a more detailed polar map was performed on the n=5-11. The Ti atom displacement vector maps show the domain evolution with changing PTO thickness. At a PTO thickness layer of 5 uc, the domain configuration reveals the polar down state. As the layer thickness increases, the domain configuration reveals a wave state around 7 uc of PTO and a vortex state around 10 uc.

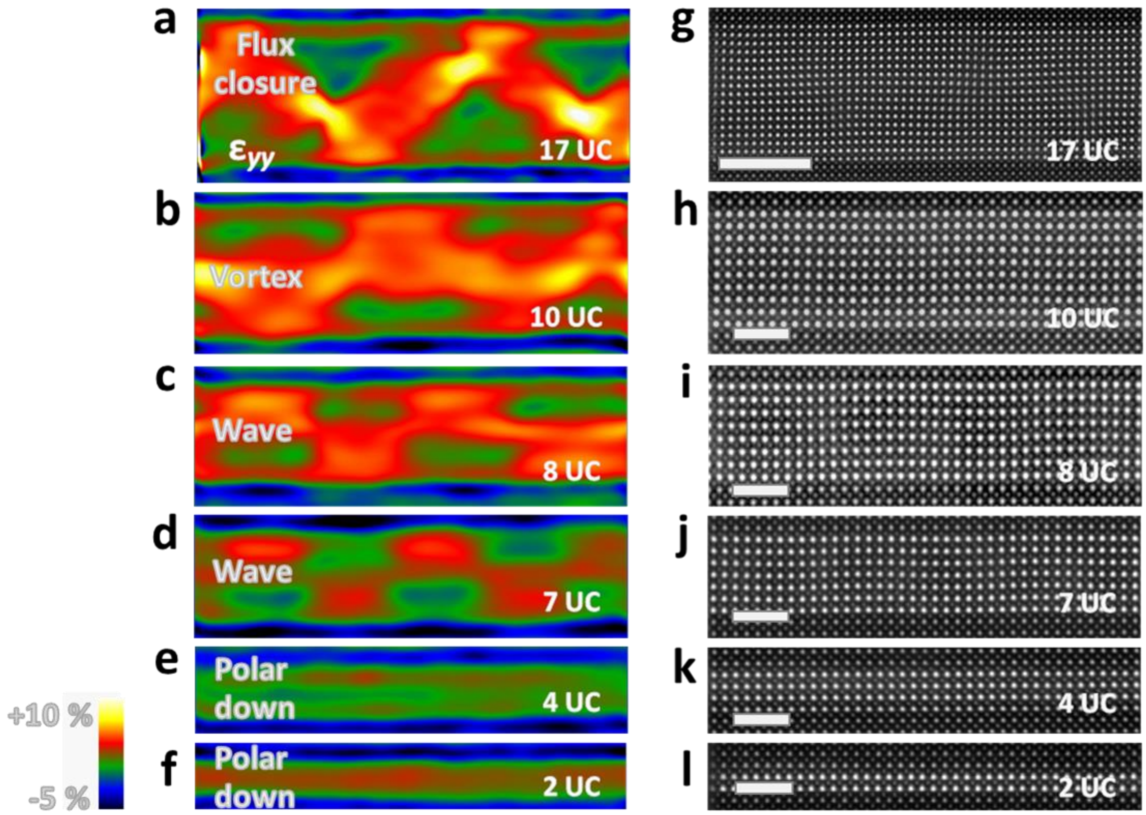

Supplementary Figure 6 Out-of-plane strain (ε_{yy}) maps together with the corresponding HAADF images. **a-f** are the GPA results extracted from different thicknesses of PTO layers, with their HAADF images in **g-l**. It is obvious that the different thicknesses of the PTO layers reveals a different intensity and pattern of the out-of-plane strain (color here represents the strength as labeled). Scale bar, 5 nm (for **g**); 2 nm (for **h-l**).

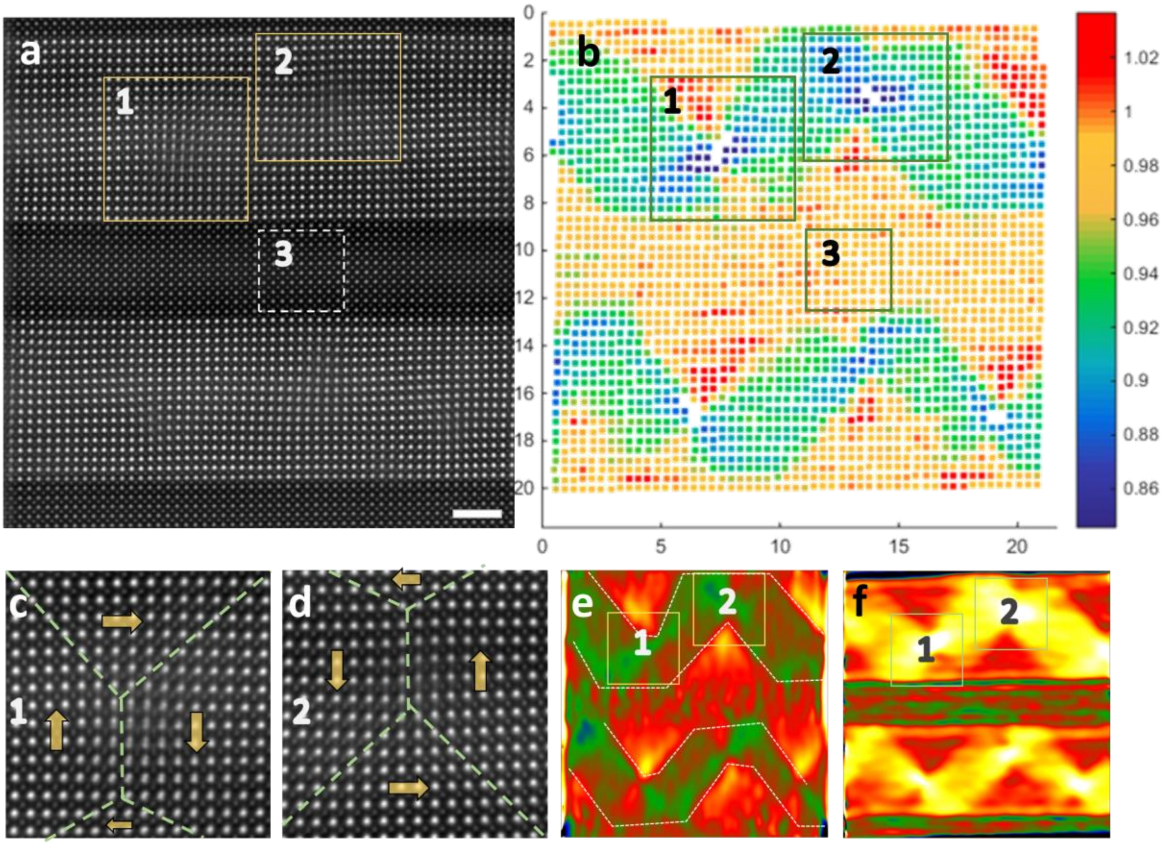

Supplementary Figure 7 HAADF-STEM images with the corresponding a/c ratio of the lattice and strain mappings. **a** HAADF-STEM image of a $PTO_{(n)}/STO_{(10)}$ multilayer, with *n*=19 and *n*=17. Boxes labeled as 1 and 2 denote representative areas. Box 3 is the reference lattice for GPA method. Scale bar, 2 nm. **b** Mapping of the a/c ratio in PTO and STO. **c** and **d** Atomically resolved HAADF-STEM images corresponding to the areas 1 and 2 in (**a**). The yellow arrows denote the polarization direction. **e** and **f** GPA analysis of the STEM data reveals that both in-plane and out-of-plane strain exhibit a sinusoidal array, which is in accordance with a/c ratio.

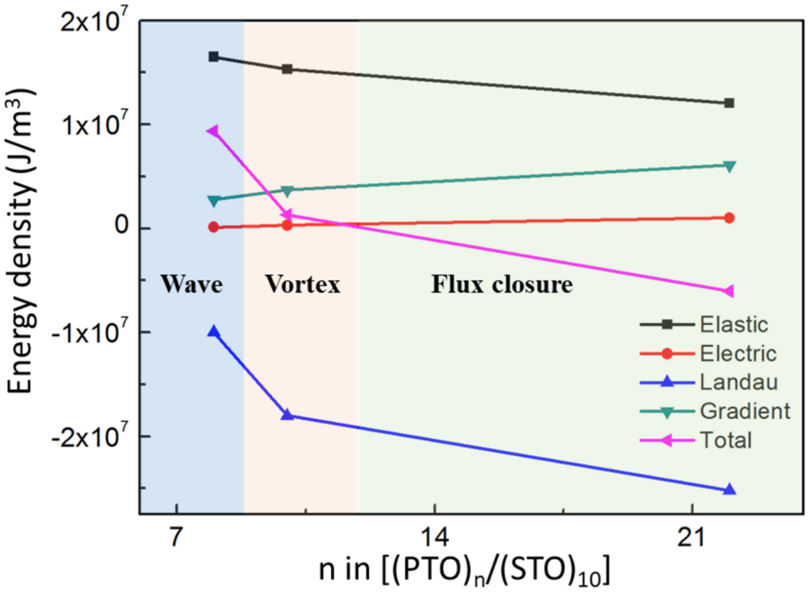

Supplementary Figure 8 The energies for PTO layers as a function of PTO thickness. The evolution of energy components of ferroelectric domain reveal the phase transition sequence with increasing PTO thickness. The decrease of the energy density of system is attributed by the drop of the average elastic and Landau energy density, which is reasonable since the flux-closure has the highest ratio of out-of-plane polarization, followed by the vortex state. Meanwhile, electric and gradient energy density increase owing to the phase transition from the wave-like state, to rotational vortex state smoothly, and to flux-closure state gradually with more distinct domain wall. Furthermore, the relatively analytical mode by Z.H et al reveals a similar tendency as experimental results[2].

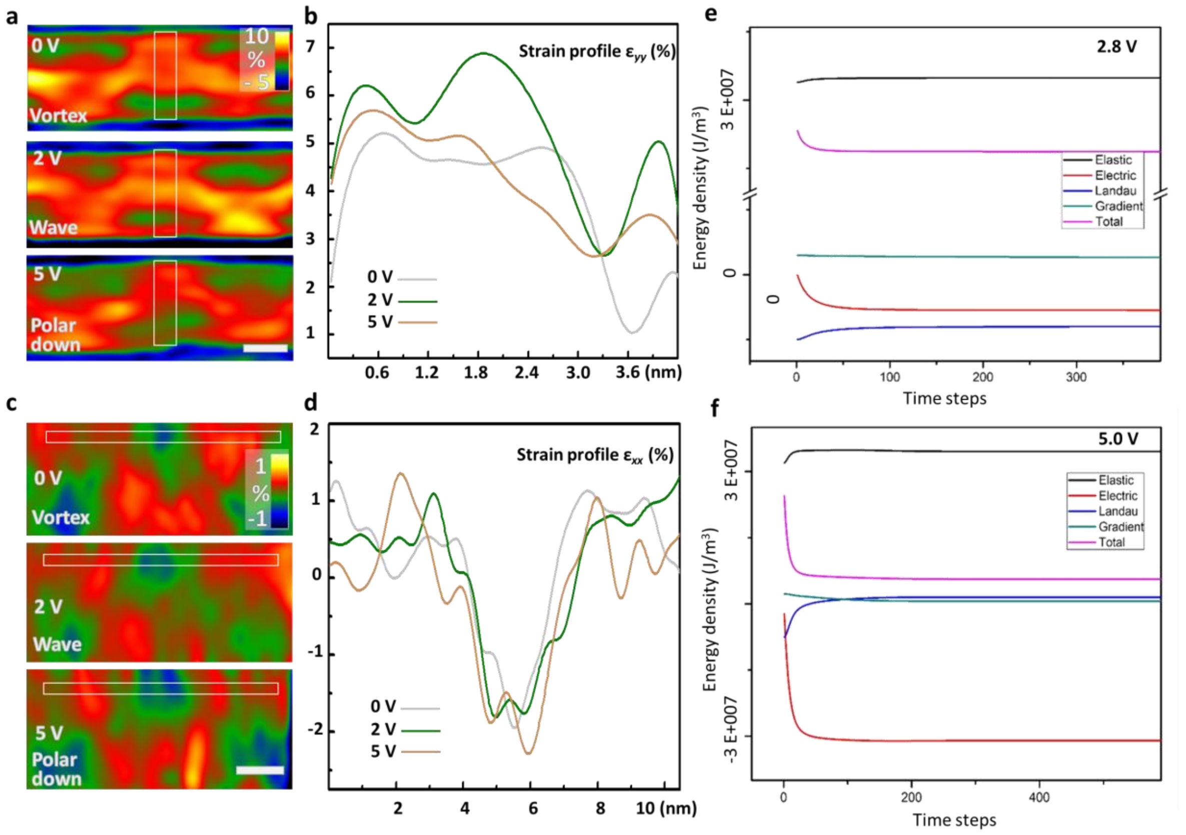

Supplementary Figure 9 Line profiles of strain in a PTO layer and time-dependent evolution of energy components of a ferroelectric domain under different bias. **a** and **c** The out-of-plane (**a**) and in-plane (**c**) strain maps of a PTO layer under an external bias of 0, 2 and 5 V. Scale bar, 2 nm. **b** and **d** Corresponding line profiles, extracted from the white boxes in (**a**) and (**c**). **e** Under a bias of 2.8 V, the reduction of electric energy compensates the increase of Landau and elastic energy, which enhances the polarization toward the external electrical field direction and reduces the polarization opposite to the field. **f** Under bias of 5 V, the large external field drives the polarization parallel to the field direction.

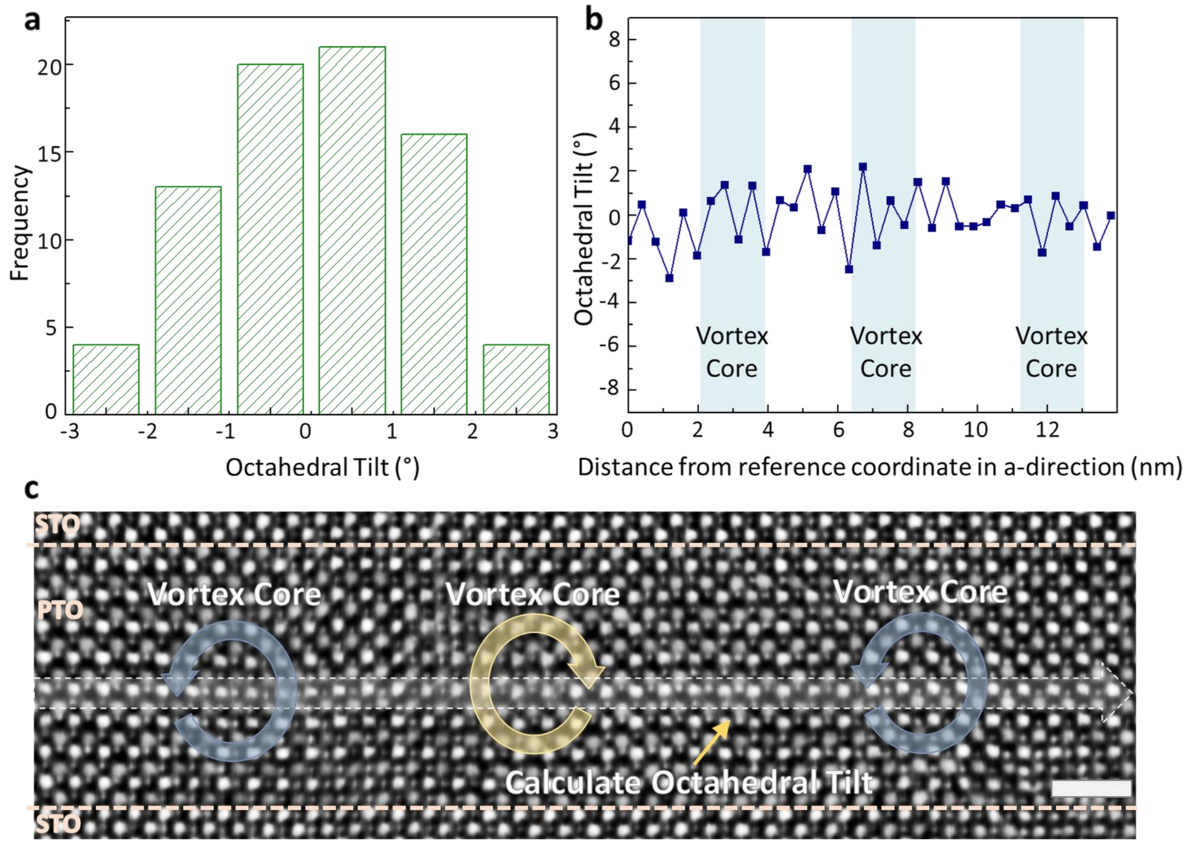

Supplementary Figure 10 Analysis of the oxygen octahedral tilt based on iDPC images. **a** Statistical results of the oxygen octahedral tilt angle. **b** Distribution of the octahedral tilt across the vortex core region. **c** the iDPC-STEM image used to calculate the octahedral tilt. From the statistical results, tilt angles were found to be below 1.5° in most unit cells of the PTO layer, which is significantly lower than the angle which is assumed to induce significant changes in electronic structure[3]. Moreover, when comparing the tilt angles in the vortex core and in non-core regions, no significant difference was observed. Scale bar, 1 nm.

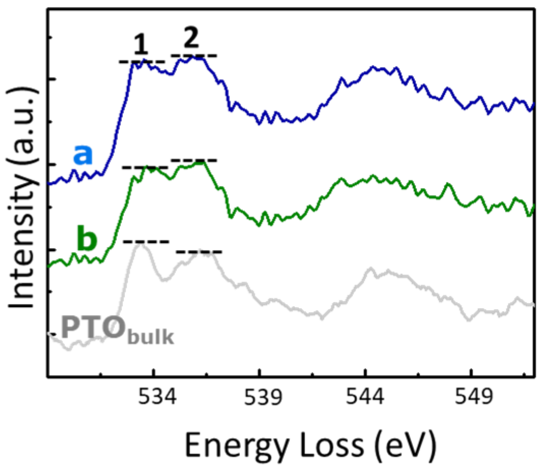

Supplementary Figure 11 EELS results of O-*K* edges in the vortex layer. The grey curve: reference O-*K* spectra acquired in the bulk-PTO; the blue a-curve was acquired in the vortex core as shown in Fig. 3 (main text); the green-b curve was acquired around the vortex cores. The peak located at ~537 eV (marked as peak 2) in the O-*K* edge is closely related to the bonding state between oxygen and the surrounding cations[4]. The grey spectrum exhibits a slightly lower peak 2 compared with the peak located at ~533 eV (marked as peak 1), in agreement with the calculated results of PTO without oxygen vacancies[5]. Differently, the blue and green spectrum exhibits an obviously enhanced peak 2, comparable to peak 1, providing evidence for the appearance of oxygen vacancies at the core-region and nearby.

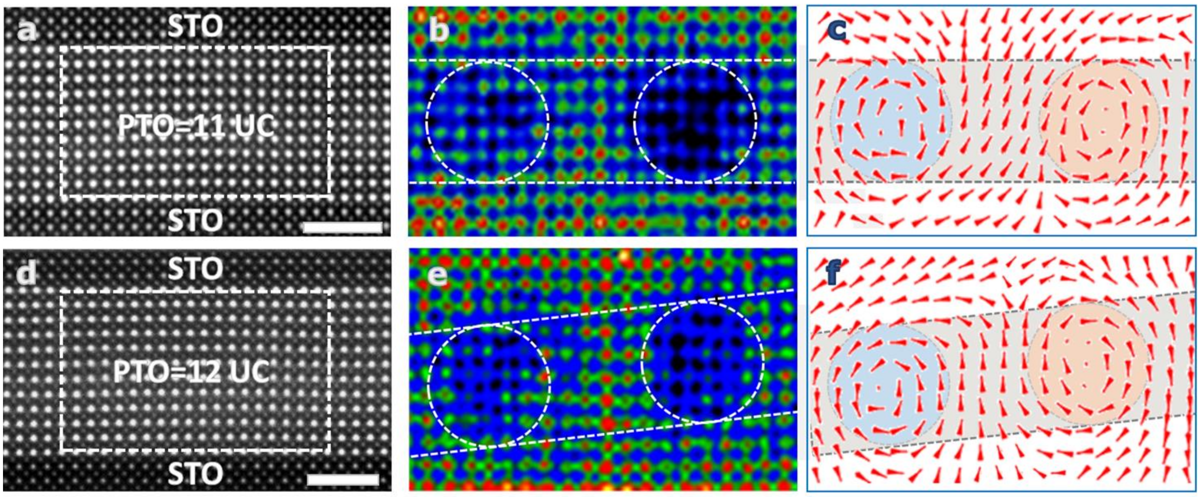

Supplementary Figure 12 EELS-mapping results in $PTO_{(11/12)}$/$STO_{(10)}$ where vortex domains exist. **a** and **d** HAADF images with the investigated areas indicated. **b**-**c** and **e**-**f** are extracted from the dashed box area in (**a**) and (**d**). **b** and **e** Ti^{4+} signal extracted from the EELS spectrum. Dashed circles represent the concentrated areas of electrons. **c** and **f** The corresponding polar vector map. It's obvious that the location of the vortex core atomically matches with the polar map. Scale bar, 2 nm.

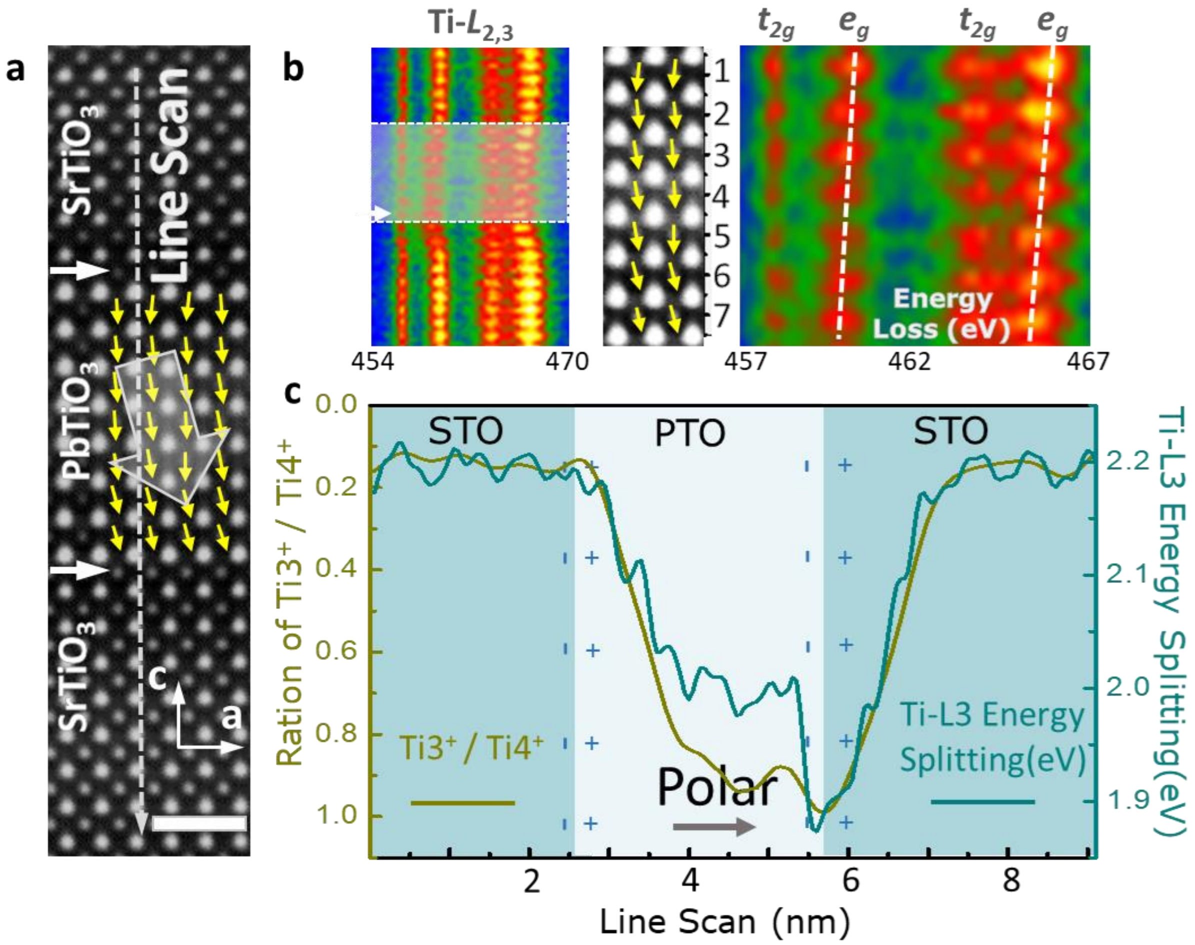

Supplementary Figure 13 EELS results in out-of-plane polarization region. **a** HAADF-STEM image of $PTO_{(8)}/STO_{(10)}$. A line scan EELS was performed across a polar down region. Scale bar, 1 nm. **b** Atomic-scale resolution Ti-$L_{2,3}$ spectra across the PTO/STO multilayer. The HAADF image is extracted from the polar down region in (**a**). Connecting the centers of the Ti-e_g peak, a distinct tilt line is observed, which means a narrowing of the energy splitting. **c** Energy splitting value as well as Ti^{3+}/Ti^{4+} ratio in the PTO and STO layers across the multilayer, which is obtained by model-based quantification of the EELS spectra and shows good agreement with the results of the Ti-L_3 energy splitting, these results indicate that the electrons is rich at the positive polar interface. The light grey area indicates the PTO layer.

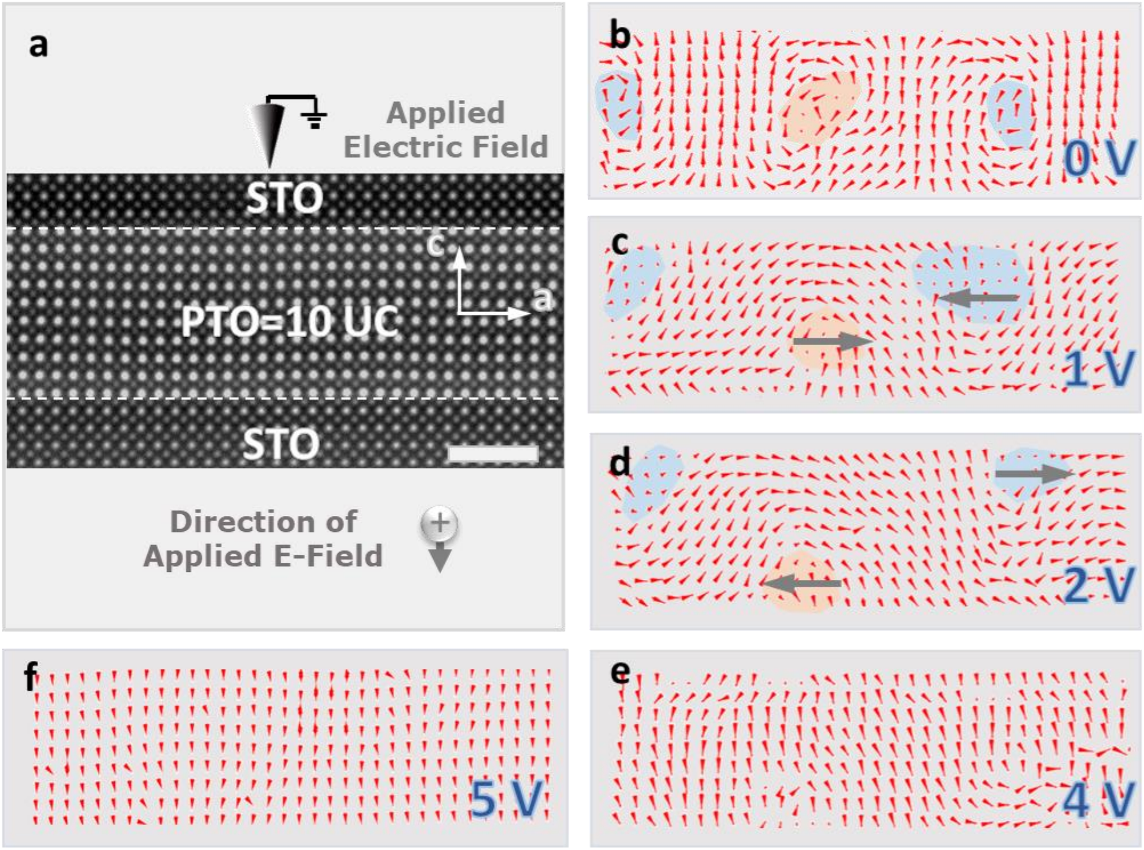

Supplementary Figure 14 Complete evolution process with applied in-situ bias. **a** and **b** Schematic diagrams of the in-situ bias experiment and the initial state. **c** Upon applying an increased voltage, the position of the vortex core reveals a zig-zag state, and then the vortex core starts to move toward the interface and they become close to each other. **d** With higher bias, the vortex cores move away from each other. **e** and **f**, Finally it becomes a polarization down state. Scale bar, 2 nm.

References

1 Yadav, A. K. *et al.* Observation of polar Vortices in oxide superlattices. *Nature* **530**, 198-201 (2016).

2 Hong, Z. *et al.* Stability of Polar Vortex Lattice in Ferroelectric Superlattices. *Nano Lett.* **17**, 2246-2252 (2017).

3 Balke, N. *et al.* Enhanced electric conductivity at ferroelectric vortex cores in $BiFeO_3$. *Nat. Phys.* **8**, 81-88 (2012).

4 Fu, L. F., Welz, S. J., Browning, N. D., Kurasawa, M. & McIntyre, P. C. Z-contrast and electron energy loss spectroscopy study of passive layer formation at ferroelectric PbTiO3/Pt interfaces. *Appl. Phys. Lett.* **87**, 262904 (2005).

5 Ryu, J., *et al.* Upshift of phase transition temperature in nanostructured $PbTiO_3$ thick film for high temperature applications. *ACS Appl. Mater. Interfaces* **6**, 11980-11987. (2014).
